# Expression of Transient Receptor Potential Vanilloid (TRPV) Channels in Different Passages of Articular Chondrocytes

**DOI:** 10.3390/ijms13044433

**Published:** 2012-04-10

**Authors:** Ismail M. Hdud, Abdelrafea A. El-Shafei, Paul Loughna, Richard Barrett-Jolley, Ali Mobasheri

**Affiliations:** 1 School of Veterinary Medicine and Science, University of Nottingham, Sutton Bonington Campus, Leicestershire LE12 5RD, UK; E-Mails: mrxih2@nottingham.ac.uk (I.M.H.); paul.loughna@nottingham.ac.uk (P.L.); 2 Department of Animal Production, Faculty of Agriculture, Al-Azhar University, Cairo, Egypt; E-Mail: dr_abdel-elshafei@hotmail.com; 3 Department of Musculoskeletal Biology, Faculty of Health and Life Sciences, University of Liverpool, Liverpool, Merseyside L69 3GA, UK; E-Mail: rbj@liverpool.ac.uk

**Keywords:** cartilage, chondrocyte, culture, passage, mechanotransduction, immunohistochemistry, immunofluorescence, dedifferentiation, TRPV4, TRPV5, TRPV6

## Abstract

Ion channels play important roles in chondrocyte mechanotransduction. The transient receptor potential vanilloid (TRPV) subfamily of ion channels consists of six members. TRPV1-4 are temperature sensitive calcium-permeable, relatively non-selective cation channels whereas TRPV5 and TRPV6 show high selectivity for calcium over other cations. In this study we investigated the effect of time in culture and passage number on the expression of TRPV4, TRPV5 and TRPV6 in articular chondrocytes isolated from equine metacarpophalangeal joints. Polyclonal antibodies raised against TRPV4, TRPV5 and TRPV6 were used to compare the expression of these channels in lysates from first expansion chondrocytes (P0) and cells from passages 1–3 (P1, P2 and P3) by western blotting. TRPV4, TRPV5 and TRPV6 were expressed in all passages examined. Immunohistochemistry and immunofluorescence confirmed the presence of these channels in sections of formalin fixed articular cartilage and monolayer cultures of methanol fixed P2 chondrocytes. TRPV5 and TRPV6 were upregulated with time and passage in culture suggesting that a shift in the phenotype of the cells in monolayer culture alters the expression of these channels. In conclusion, several TRPV channels are likely to be involved in calcium signaling and homeostasis in chondrocytes.

## 1. Introduction

Articular cartilage is a unique and mechanically resilient connective tissue that experiences a variety of stresses, strains and loading pressures during physical activity and locomotion [1]. Chondrocytes are specialized cells responsible for the synthesis, maintenance and degradation of the cartilage extracellular matrix (ECM) [2]. The cartilage ECM consists primarily of type II collagen and aggregating proteoglycans, which give the tissue the ability to resist tensile stress and physical load respectively [3]. Chondrocytes have the capacity to detect and respond to mechanical loads by altering their metabolic state through a process known as mechanotransduction [4]. Mechanotransduction is a series of dynamic processes that allow living cells to convert biomechanical stimuli into biochemical signals. In cartilage, mechanotransduction represents the cellular and extracellular mechanisms by which chondrocytes modulate the rates of matrix synthesis and degradation and alter the composition of the ECM. Mechano-electrochemical responses of chondrocytes under mechanical load involve changes in osmotic pressure and the electrical membrane potential difference across the chondrocyte plasma membrane [5]. Chondrocytes sense biomechanical, ionic, osmotic and electrical signals and respond to these varied signals in coordination with other environmental, hormonal and genetic factors to regulate metabolic activity [6]. Changes in ionic and osmotic pressure, ion transport, fluid flow and electrical current across the chondrocyte membrane are important mechano-electrochemical phenomena in cartilage under mechanical load.

The mechanisms involved in mechanotransduction in chondrocytes are poorly understood. Mechanically induced cell membrane deformation is one of a number of possible mechanotransduction pathways by which chondrocytes sense and respond to mechanical changes in their environment [7–9]. A number of ion channels, including transient receptor potential channels (TRP), have been implicated in mechano-electrochemical coupling in chondrocytes [10]. The mammalian TRP protein comprises a superfamily of more than 28 members of ion channels that display variability in their permeation properties with preference for calcium ions [11,12]. The transient receptor potential vanilloid (TRPV) subfamily comprises of six members that have been implicated in nociception, thermo and osmosensing (TRPV1-4) [13,14]. Some of the TRPV channels are highly selective for Ca^2+^ over other cations and are involved in Ca^2+^ absorption/resorption in the gut and kidney (TRPV5-6) [15,16]. In canine chondrocytes, TRPV5 also has a role in setting the membrane potential [17]. TRPV4 was one of the first osmotically sensitive ion channels to be identified in vertebrates [13,14]. It is permeant to several cations with a preference for Ca^2+^ [18,19] and is activated in response to hypo-osmotic stress [18,20], heat [21], phorbol esters such as 4α-phorbol 12,13-didecanoate [22], arachidonic acid (AA) and its metabolites [23]. TRPV4^−/−^ knockout mice show decreased responsiveness to systemic osmotic stress and sensation of noxious mechanical stimuli [24,25]. These studies have demonstrated a role for TRPV4 in the regulation of cell volume recovery following hypo-osmotic stress through Ca^2+^ signaling. Recent studies have demonstrated the presence of functional TRPV4 channels in porcine and murine articular cartilage [10,26].

Changes in ion channel expression and activity play important roles in cellular signal transduction. Accordingly, the aim of this study was to investigate the effect of time in culture and passage on expression of TRPV4, TRPV5 and TRPV6 in equine articular chondrocytes. Western blotting was used to examine and quantify the expression of TRPV4, TRPV5 and TRPV6 in first expansion (P0) and serial passages (P1–3) of equine articular chondrocytes. The tissue distribution of these channels was investigated in sections of formalin fixed paraffin embedded equine articular cartilage using immunohistochemistry. Immunofluorescence was used to study the cellular and subcellular localization of the channels in monolayer cultures of methanol fixed P2 chondrocytes.

## 2. Results

### 2.1. Immunohistochemical Distribution of TRPV Channel Isoforms in Sections of Equine Articular Cartilage and in Chondrocyte Cultures

**Figure 1:**
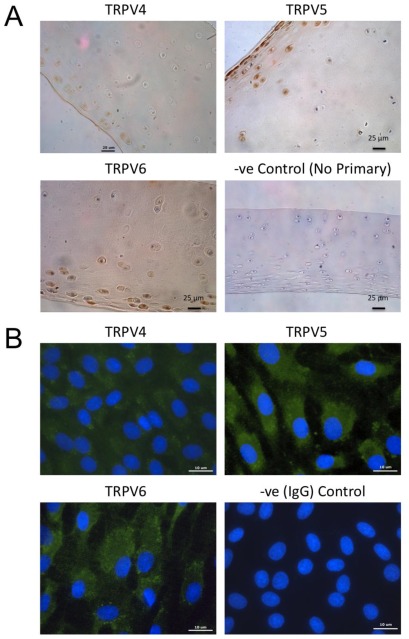
(**A**) Localization of transient receptor potential vanilloid (TRPV) channel isoforms (TRPV4, TRPV5 and TRPV6) in equine articular cartilage. Sections of equine articular cartilage were immunostained using polyclonal antibodies raised against TRPV4, TRPV5 or TRPV6. All three channels showed positive immunoreactivity in chondrocytes from the superficial zone of equine articular cartilage. The negative control shown was treated using the same immunohistochemical protocol, but the primary antibody step was omitted; (**B**) Cellular and subcellular localization of TRPV4, TRPV5 and TRPV6 in monolayer cultures of methanol fixed P2 chondrocytes. All three channels showed positive immunoreactivity (green fluorescent staining) within P2 chondrocytes. The negative control was exposed to nonimmune rabbit IgG and shows blue fluorescent nuclear staining only. Nuclei were counterstained with DAPI (blue fluorescent staining).

We investigated the localization of TRPV channel isoforms within equine articular cartilage by immunohistochemistry using affinity purified polyclonal antibodies raised against TRPV4, TRPV5 and TRPV6. A high level of expression was observed in chondrocytes in sections of macroscopically and microscopically normal equine articular cartilage. Immunostaining was detected in chondrocytes in the superficial and middle zones of equine articular cartilage. The intensity of the immunostaining was much lower in chondrocytes of the deep zone (Figure 1A). Negative controls involved omitting primary antibodies and did not show evidence for any non-specific immunostaining for TRPV4, TRPV5 and TRPV6 within equine articular chondrocytes. Immunofluorescence was also used to study the expression and subcellular distribution of TRPV4, TRPV5 and TRPV6 in monolayer cultures of P2 chondrocytes (Figure 1B).

### 2.2. Expression of TRPV Channel Isoforms in Passaged Chondrocytes

**Figure 2:**
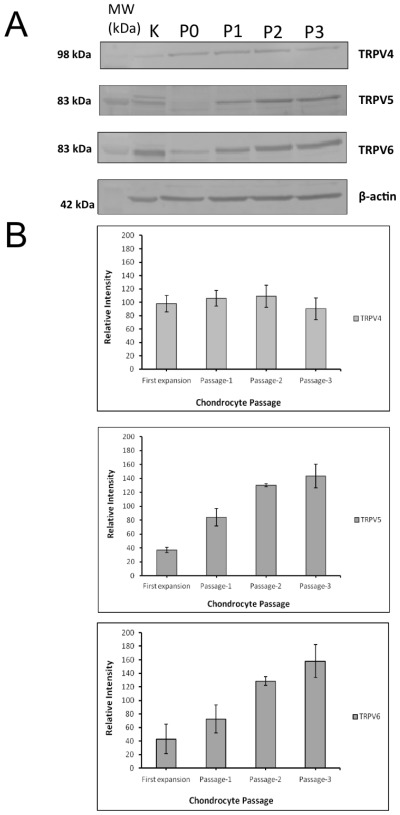
(**A**) Western blot analysis of total chondrocytes lysate (25 μg/lane) from first expansion (P0) and serial passages (P) 1–3. Kidney was used as a positive control. Western blotting confirmed the expression of TRPV4, TRPV5 and TRPV6 proteins in different passages. B. Image analysis of western blots. No significant change in expression profile was observed in TRPV4 among different passages. TRPV5 and TRPV6 expression was significantly upregulated with time and passage in culture (*P* < 0.05).

Expression of TRPV channel proteins (TRPV4, TRPV5 and TRPV6) was examined using western blots of freshly isolated equine articular chondrocytes (P0). Expression of β-actin (an abundant housekeeping protein) was used as an internal loading control. Total protein extracted from equine kidney was used as a positive control. The western blotting detection of TRPV4, TRPV5 and TRPV6 was carried out as described in several recent publications [27,28]. The molecular weights calculated in previous studies were 101–107 kDa for TRPV4 and, 85–100 kDa for both TRPV5, TRPV6. In this study, the immunoreactive bands were determined to be approximately 98 kDa for TRPV4, 83 kDa for both TRPV5 and TRPV6 (Figure 2A).

### 2.3. Expression Profile of TRPV Channels in Chondrocytes Changes with Serial Passage

Chondrocyte lysates were collected from different passages including P0, and serial passages 1–3 (P1–3). Proteins were separated by SDS-PAGE and electroblotted as described in the Experimental Section. Quantitative differences in the expression of TRPV channel proteins were calculated by densitometric analysis of western blots. The relative intensity of immunoreactive bands for each TRPV channel from each passage was quantified using ImageJ (Image Processing and Analysis in Java; http://rsb.info.nih.gov/ij/). Expression ratios are reported as mean ± the standard deviation. Densitometric analysis revealed no significant changes in the expression profile of TRPV4 channel across all passages. However the expression of both TRPV5 and TRPV6 channels was upregulated with time in culture and passage number (Figure 2B). The expression of TRPV5 channel was increased two-fold at P1 and three-fold at P2 and P3 compared to first expansion (P0) cells. TRPV6 expression was also upregulated two-fold at P1, three-fold at P2 and almost four-fold at P3 relative to the P0 cells. This may be related to the gradual de-differentiation of the chondrocytes as they progress from P0 to P3, as the cells lose their phenotype and become more fibroblastic in monolayer culture.

## 3. Discussion

In this study western blot analysis was employed to demonstrate the expression of transient receptor potential vanilloid channel members 4, 5 and 6 (TRPV4, TRPV5 and TRPV6) in equine articular chondrocytes. Furthermore, we compared the expression levels of these channels in serially passaged articular chondrocytes. Serial monolayer culture results in chondrocyte dedifferentiation and loss of phenotype. Isolated primary chondrocytes do not maintain their phenotype when cultured for a prolonged period. They have the tendency to dedifferentiate to a fibroblastic phenotype after four or more passages [29,30]. When chondrocytes dedifferentiate in monolayer culture they decrease proteoglycan synthesis and switch to production of type I collagen instead of type II. Therefore, in this study, freshly isolated primary equine articular chondrocytes were used and the passage number was not allowed to exceed three. Despite this, the expression of TRPV5 and TRPV6 was significantly upregulated during the course of the study.

Although chondrocytes seem to exhibit similar signaling responses to osmotic stress both *in situ* and *in vitro* [31], recent reports suggest that chondrocyte interactions with the ECM are also involved in signal transduction. We therefore investigated the expression of TRPV4, 5 and 6 in sections of cartilage. Immunohistochemical analysis revealed the presence of each of these proteins in chondrocytes from both superficial and middle zone cartilage. This observation is entirely consistent with previous studies which show the presence of TRPV4 in chondrocytes *in situ* in human [32], mouse [10], bovine [33] and porcine [26] cartilage.

The role of ion channels, especially Ca^2+^ channels in chondrocyte biology has been an area of intense research. Intracellular Ca^2+^ controls many cellular functions including transcriptional regulation, migration and proliferation [32]. Ca^2+^ channels have also been reported to influence chondrocyte metabolism and chondrocyte differentiation [34]. For example increasing extracellular Ca^2+^ concentration in cell culture promotes chondrocyte de-differentiation whereas decreasing extracellular Ca^2+^ increases collagen biosynthesis of proteoglycans and delays hypertrophy [35].

The physiological roles of ion channels in chondrocytes are gradually becoming established [36]. There is increasing interest in TRPV channels in these cells in the context of volume homeostasis. Recent studies have demonstrated a role for TRPV4 in the regulation of cell volume in several cell types including chondrocytes [5]. Inhibition of this channel using the specific pharmacological inhibitor (GSK205) prevents cells from responding to hypo-osmotic stress by normal regulatory volume decrease (RVD) [26]. Therefore TRPV4 activation [10] together with activation of calcium activated potassium channels [37,38] appear to be central to the process of osmoregulation and mechanotransduction in chondrocytes.

TRPV5 expression has been reported in the duodenum, kidney and heart, where it is thought to be involved in Ca^2+^ absorption, re-absorption and cardiomyocyte contraction. TRPV5 and TRPV6 are thought to be responsible for calcium absorption; TRPV5 in kidney [39] and TRPV6 in the duodenum [40] although co-expression of both channels has been reported in both tissues. The roles TRPV5 and TRPV6 in chondrocytes are gradually being unraveled; evidence suggests that they contribute to setting the membrane potential [17]. Interestingly, however, despite the upregulation of TRPV5/6 with early passage of chondrocytes, there is no significant change in membrane potential over this period [17]. Recent work conducted by Gavenis and co-workers have reported the elevation of gene expression for members of TRPC (Canonical) (TRPC3 and TRPC2) in chondrocytes after passage two in culture [32]. This study has certain similarities with our TRPV5 and TRPV6 results suggesting that serial chondrocyte passage can affect the expression of a number of ion channels in chondrocytes. Therefore, it would be interesting to see if such changes occur in osteoarthritis or with cartilage ageing.

## 4. Experimental Section

### 4.1. Articular Cartilage

Normal (healthy) equine joints (*n* = 3 horses) were obtained from the abattoir (Taunton, Somerset, UK). Articular cartilage was dissected from metacarpophalangeal joints of mature horses euthanized for unrelated clinical reasons. In co-ordination with national guidelines, ethical and institutional approval was obtained before sample collection.

### 4.2. Histology and Tissue Preparation

Full depth chips/samples of equine articular cartilage were fixed in 10% neutral buffered formalin (NBF) for 24 h and decalcified in 10% ethylenediaminetetraacetic acid (EDTA) for 8 weeks. Cartilage samples were dehydrated in increasing concentrations of ethanol (30%, 50%, 70%, 90%, 95% and 100%) at room temperature. Samples were embedded in paraffin wax, sectioned 5 μm and mounted on super frost ultra slides (Thermo scientific, UK). Morphological assessments were performed using hematoxylin and eosin stain. Stained slides were examined with a Leica DM 5000B microscope.

### 4.3. Chondrocyte Isolation and Culture

Chondrocytes were isolated from equine cartilage using type I collagenase from *Clostridium histolyticum* (≥125 collagen digestion units/mg solid; C0130, Sigma-Aldrich, UK) as described previously [17,41,42]. Cartilage shavings were digested with a freshly prepared solution of 0.1% type I collagenase in free serum DMEM for 18 h at 37 °C. At the end of the digestion period any remaining undigested cartilage fragments were removed by filtering the supernatant through a 70 μm nylon cell strainer (BD Bioscience, Europe). The filtered mixture was centrifuged, the supernatant discarded and the chondrocytes resuspended in phosphate buffered saline (PBS) supplemented with 10% penicillin/streptomycin (Invitrogen, Paisley, UK) and washed three times. Finally, the cells were resuspended in low glucose DMEM supplemented with 2% penicillin/streptomycin and 10% FCS and cultured in T75 flasks at 37 °C, 5% CO_2_. The isolated chondrocytes were either directly used for investigation (first expansion) (P0) or grown in monolayer until confluence was reached (passages (P) 1–3). P1–3 cells were obtained by detaching chondrocytes from flasks using Trypsin/EDTA (Invitrogen) digestion (0.05%/0.02% in PBS). Chondrocytes were counted and either used in experiments for investigation of passage (P1) or cultivated in monolayer in a new T75 flask. The same process was repeated for P2 and P3.

### 4.4. Antibodies

Polyclonal isoform specific antibodies raised in rabbits against TRPV channel isoforms were purchased from Abcam Plc (Cambridge, UK). The following antibodies and codes were used: TRPV4 (ab39260), TRPV5 (ab63085) and TRPV6 (ab63084). A polyclonal antibody against β-actin was obtained from Sigma Aldrich, UK (code A5316).

### 4.5. Immunohistochemistry

Sections of equine articular cartilage were probed for the expression of TRPV channel isoforms (TRPV4, TRPV5 and TRPV6) utilizing immunohistochemistry as previously described in several relevant publications [38,41,42]. Briefly, formalin fixed, paraffin embedded articular cartilage sections (5μm in thickness) were dewaxed in xylene for 10 min to remove embedding medium, rehydrated in a descending gradient of ethanol solutions (100%, 95%, 90%, 70% 50% and 30%) for 2 min each and placed in water for 5 min. Activity of endogenous peroxidase was blocked by incubating in 3% (v/v) hydrogen peroxide diluted in PBS buffer for 30 min, followed by blocking of non-specific antibody binding in PBS buffer with 0.05 Tween-20 (PBS-T) and 1% protease free bovine serum albumin (BSA) (Fisher Scientific, UK) for 1 h at room temperature. Overnight incubations with antibodies to TRPV channels were performed at 4 °C according to the manufacturer’s recommended dilutions in PBS-T (antibodies were typically diluted 1:200). The following day the slides were washed 3 times for 5 min each (with agitation) with PBS-T and then incubated with horseradish peroxidase (HRP) labeled polymer conjugated to affinity purified goat anti-rabbit immunoglobulin (code K4065, Dako, UK) for 1 h at room temperature. After 3 washes with PBS-T for 5 min each, the sections were incubated with liquid DAB+ chromogen (code SK4100, Vector Laboratories). The development of the brown colored reaction was closely monitored under the light microscope and immersing the slides in distilled water terminated the reaction. Cell nuclei were counterstained by immersing the slides in an aqueous hematoxylin bath (VWR international Ltd) for 2 min followed by washing in running water for 5 min and dehydration in an ascending gradient baths of ethanol solutions (50%, 70%, 90%, 95% and 100%) for 2 min each. Finally the slides were rinsed in two fresh xylene baths for 5 min each before mounting in 1, 3-diethyl-8-phenlxanthine (DPX) (Fluka analytical, Sigma-Aldrich, UK). Negative controls involved exclusion of the primary antibody from the immunohistochemical procedure.

### 4.6. Immunofluorescence

Briefly, P2 chondrocytes were grown in DMEM on autoclaved coverslips at a density of 3 × 10^5^ cells/mL. They were then fixed in ice-cold methanol for 10 min, washed and permeabilized with PBS-T and blocked with 10% BSA in PBS. The cells were then incubated with the primary antibodies to TRPV4, TRPV5 and TRPV6 overnight as described in the immunohistochemistry section above. After 3 washes with PBS-T the cells were incubated for 2 hours with a goat polyclonal secondary antibody to rabbit IgG (Fc-specific, affinity purified, pre-adsorbed) conjugated to DyLight® 488 (Abcam ab98462) diluted according to the manufacturer’s recommendations (typically diluted 1:200). After extensive washes in PBS-T the coverslips were mounted using Prolong Gold anti-fade reagent with 4′,6-diamidino-2-phenylindole (DAPI) (Invitrogen, P36935). The cells were visualized and digital images were captured using a Leica DM 5000B epifluorescence imaging system.

### 4.7. Western Blotting

Chondrocytes from P0 and serial passages P1–3 were washed three times with sterile PBS. Total protein extraction was carried out by lysis in radio-immunopreciptation assay (RIPA) buffer (150 mM NaCl, 50 mM Tris-HCl, pH 7.5, 5 mM ethylene glycol tetraacetic acid (EGTA), 1% Triton, 0.5% sodium deoxycholate and 0.1% sodium dodecyl sulphate (SDS)) supplemented with phosphatase and protease inhibitor cocktail (Roche Diagnostic, Mannheim, Germany) on ice for 30 min. Cells debris was removed by centrifugation at 14,000 rpm for 10 min. The protein concentration of the supernatant was determined by using the Bradford assay with BSA as the standard [43]. Supernatants were mixed with 4× sample buffer (0.5 M Tris-HCl, pH 6.8, 100% glycerol, 20% SDS, 0.5% bromophenol blue and 5% β-mercaptoethanol) and denatured at 90 °C for 3 min. Total protein extracts (25 μg) were separated under denaturing conditions by SDS polyacrylamide gel electrophoresis using a 4% stacking gel and a 10% resolving, then transferred to polyvinylidene difluoride PVDF membrane (Invitrogen) utilizing a semi-dry electroblotting apparatus (Bio-Rad, UK). Blots were blocked in 5% (w/v) fat-free skimmed milk (Marvel®) in TBS/0.1% Tween-20 for 1 h at room temperature. Membranes were probed with primary antibodies to TRPV4 (1:200), TRPV5 (1:200) and TRPV6 (1:200) diluted in blocking buffer overnight at 4 °C. After five washes in TBS/0.1% Tween-20, membranes were incubated with goat anti-rabbit IgG conjugated with horseradish peroxidase (HRP) (Dako, UK) secondary antibody for 1 h at room temperature. Membranes were washed five times for 5 min each in TBS/0.1% Tween-20, and developed using Amersham ECL western blot enhanced chemiluminescence kit (GE Healthcare, UK).

## 5. Conclusions

This study has demonstrated that the TRPV4, TRPV5 and TRPV6 channels are present in equine articular chondrocytes *in situ* as well as in isolated and passaged cells. TRPV4 may act as an osmosensor and regulate chondrocyte responses to changes in joint loading. Our results indicate an association between the degree of chondrocyte de-differentiation and the expression of TRPV5 and TRPV6. Further studies are needed to determine whether other TRP superfamily members are affected by passage and de-differentiation in chondrocytes. Biological, physical and pharmacological manipulation of these channels may influence chondrocyte de-differentiation and reveal new strategies for maintaining their phenotype for autologous chondrocyte implantation (ACI) and cartilage tissue engineering. This information will also further our understanding of the relationship between the level of expression of TRPV channel isoforms and osmotic/mechanical stress changes in the joint and may provide new insight into the process of mechanotransduction and the compromised biomechanical mechanisms that are thought to contribute to the pathogenesis of osteoarthritis.
